# Effects of hexafluoro-2-propanol on inflammatory and hemodynamic responses in a rat model of endotoxic shock

**DOI:** 10.1186/cc10626

**Published:** 2012-03-20

**Authors:** M Urner, IK Herrmann, M Hasler, C Booy, B Beck-Schimmer

**Affiliations:** 1 University Hospital Zurich, Institute of Anesthesiology, Zurich, Switzerland

## Introduction

Sepsis with multiple organ failure remains a leading cause of hospital morbidity and mortality on ICUs imparting tremendous financial costs. Recently, the primary metabolite of sevoflurane, hexafluoro-2-propanol (HFIP), has been found to exert immunomodulatory properties attenuating inflammatory response to lipopolysaccharides (LPS) *in vitro *[1]. We investigated whether HFIP attenuates plasma and tissue inflammatory mediator expression in a rat model of endotoxic shock.

## Methods

Thirty-two male Wistar rats were anesthetized, tracheotomized, and mechanically ventilated. The animals were randomly assigned to one of the following groups: (I) LPS group (*n *= 8), which received intravenous *Escherichia coli *endotoxin (1 mg/kg); (II) LPS/HFIP group (*n *= 8), which was treated identically to the LPS group with the additional administration of HFIP (67 μg/kg over 30 minutes) after LPS injection. Control groups received Ringer's lactate instead of LPS. General anesthesia was maintained with propofol. All animals received additional 30 ml/kg Ringer's lactate after injection of LPS over a time period of 1 hour. Arterial blood gases were measured every hour. Animals were euthanized 6 hours after endotoxin injection. The concentrations of monocyte chemoattractant protein-1, key player in the recruitment of monocytes during endotoxemia, was analyzed in bronchoalveolar lavage fluid and in plasma. Linear regression was used to evaluate influence of HFIP on inflammatory mediator expression.

## Results

Plasma MCP-1 protein levels assessed 6 hours after LPS injection were increased by +5,192 ng/ml compared to baseline (*R*^2 ^= 0.661, *P *< 0.001). This increase in MCP-1 protein was attenuated by -48% in the LPS/HFIP group (+2,706 ng/ml to baseline, *R*^2 ^= 0.661; *P *= 0.004). Similar results were found in BALF, in which HFIP decreased the LPS-induced raise in MCP-1 protein concentration by -62% (difference of 54 ng/ml, *P *= 0.034). LPS-stimulated animals had a +12% higher mean arterial blood pressure after 6 hours when treated with HFIP (78 mmHg vs. 67 mmHg, *R*^2 ^= 0.684, *P *= 0.035). No significant differences in lactate levels were observed. HFIP attenuated base deficit in LPS-stimulated animals by 1 mmol/l (*R*^2 ^= 0.522, *P *= 0.034).

## Conclusion

Hexafluoro-2-propanol attenuated LPS-induced inflammatory mediator secretion, the decrease in mean arterial blood pressure, and base deficit. These results suggest that hexafluoro-2-propanol may partly inhibit inflammatory response, hypotension and the development of metabolic acidosis during endotoxic shock.
